# Comprehensive Strategy for Sample Preparation for the Analysis of Food Contaminants and Residues by GC–MS/MS: A Review of Recent Research Trends

**DOI:** 10.3390/foods10102473

**Published:** 2021-10-15

**Authors:** Meng-Lei Xu, Yu Gao, Xiao Wang, Xiao Xia Han, Bing Zhao

**Affiliations:** 1 State Key Laboratory of Supramolecular Structure and Materials, College of Chemistry, Jilin University, Changchun 130012, China; xumenglei@jlu.edu.cn; 2 College of Food Science and Engineering, Jilin University, Changchun 130062, China; 3 College of Plant Protection, Jilin Agricultural University, Changchun 130118, China; gaothrips@jlau.edu.cn; 4 Jilin Institute for Food Control, Changchun 130103, China; wangxiao3777@gmail.com

**Keywords:** GC‒MS/MS, pretreatment methods, food contaminants, QuEChERs, adsorption extraction

## Abstract

Food safety and quality have been gaining increasing attention in recent years. Gas chromatography coupled to tandem mass spectrometry (GC–MS/MS), a highly sensitive technique, is gradually being preferred to GC–MS in food safety laboratories since it provides a greater degree of separation on contaminants. In the analysis of food contaminants, sample preparation steps are crucial. The extraction of multiple target analytes simultaneously has become a new trend. Thus, multi-residue analytical methods, such as QuEChERs and adsorption extraction, are fast, simple, cheap, effective, robust, and safe. The number of microorganic contaminants has been increasing worldwide in recent years and are considered contaminants of emerging concern. High separation in MS/MS might be, in certain cases, favored to sample preparation selectivity. The ideal sample extraction procedure and purification method should take into account the contaminants of interest. Moreover, these methods should cooperate with high-resolution MS, and other sensitive full scan MSs that can produce a more comprehensive detection of contaminants in foods. In this review, we discuss the most recent trends in preparation methods for highly effective detection and analysis of food contaminants, which can be considered tools in the control of food quality and safety.

## 1. Introduction

Since the 1970s, gas chromatography (GC) coupled to mass spectrometry (MS) has been applied to the detection of most contaminants and residues routinely found in foods. However, in the last ten years, the majority of food control laboratories moved from GC–MS to GC–MS/MS as the preferred analytical technique to address GC amenable compounds, mainly due to the interference in single-step GC–MS analysis caused by coeluting matrix compounds. Certain compounds cannot be separated in single-step MS because they coincide with selected ions in GC–MS. In order to analyze multiple compounds by GC–MS, it is necessary to ensure that all target compounds can be adequately separated qualitatively and quantitatively.

A sample preparatory step in food contaminants detection can be included to enable high recovery and good reproducibility, which should ideally be rapid, inexpensive, simple, easy to automatize, and environmentally friendly. High separation in MS/MS may substitute the importance of preparation selectivity; however, the choice of the method for the sample preparatory step is also determined by the type of food matrix as well as by the contaminants of interest. Thus, multi-residue preparation methods, such as QuEChERs and adsorption extraction, are a most important technology enabling the simultaneous extraction of as many targets as possible [1]. When a large number of contaminants is to be detected in samples, MS/MS must operate in multi-reaction monitoring (MRM) mode, and the use of multi-residue analysis methods in analysis providing high sensitivity and high specificity has become the new trend.

Among emerging chemical hazards in foods, contaminants that may constitute a threat to human health and the environment in the future can be included. These hazards might include not only contaminants of emerging concern, such as brominated flame retardants (BFRs), perfluorochemicals (PFOS), endocrine disrupters (ERs), but also those of biological origin. Thus, the ideal sample extraction procedure and purification method should take into account the contaminants of interest. Moreover, these methods should cooperate with high-resolution MS, and other sensitive full scan MSs that can produce a more comprehensive detection of contaminants in foods.

GC–MS/MS has become a major technique for the analysis of contaminants and residues in foods due to their high sensitivity and selectivity, being widely used for the analysis of low-polarity, volatile, and thermally stable compounds. Considering the nature of contaminants detected in foods, we summarized herein and discussed two commonly used processes: (1) analysis of volatile organic compounds (VOCs) by headspace (HS) injection with/without derivatization; (2) and analysis of semi-volatile organic compounds (SVOCs) or thermally stable compounds after extraction and clean-up. In general, foods, such as grains, vegetables, fruits, sugars, beverages, edible fungi, flavorings, medicinal plants, and foods of animal origins, are often complex matrices. Moreover, foods can be classified according to their form into solid and liquid food matrices, and effective analytical strategies must take into account the type of food matrix.

The aim of this review article was to discuss preparation methods for the analysis of contaminants and residue in foods by GC–MS/MS with an emphasis on literature published in recent years. Promising future trends and perspectives are also discussed.

## 2. Preparation Methods for the Analysis of VOCs

VOCs in food contaminants mainly include phthalate esters (PAEs), polycyclic aromatic hydrocarbons (PAHs), polychlorinated biphenyls (PCBs), aldehydes, and certain pesticides. In order to achieve a practical and reliable method for the determination of VOCs in food samples, several preparation methods have been developed, such as HS extraction and solid-phase microextraction (SPME).

### 2.1. HS Extraction

HS extraction is a sample preparation procedure that has demonstrated its usefulness for a broad range of VOCs in the headspace, which has been shown to reduce interference of the matrix for food substrates [2]. HS extraction is chiefly based on the adsorption of analytes on fiber coating. After establishing equilibrium between the HS of the sample and the fiber coating, components are desorbed from the fiber into a chromatography column. HS methods can be divided into static HS and dynamic HS (DHS) extraction. Static HS sampling is a conventional sample preparation method used for the analysis of VOCs from herbs and foods. It is a rapid and solvent-free method that requires only a small aliquot of samples [3]. DHS extraction can be performed by continuously sweeping the HS of the sample with a significant quantity of gas. Then, the extracted gas is loaded onto a selective adsorbent where analytes are trapped. Thermal desorption of trapped analytes is then required before conducting cryofocus GC–MS analysis. This approach has already been used to determine VOCs in fish and wine as well as to characterize olive oil [4,5,6].

### 2.2. Solid-Phase Microextraction (SPME)

SPME has been used for sample preparation of a wide range of foods due to its sensitivity and convenience of quantitation [7]. SPME can be regarded as a short GC column turned inside out. A fiber coating can be used as a filter to extract chemicals from different samples. SPME has the advantages of being simple to operate, highly efficient, solvent-free, and employed reagents can be reused. SPME can combine sampling, extraction, pre-enrichment, and injection into one step. Nowadays, commercial SPME fiber coatings, such as polydimethylsiloxane (PDMS) and PDMS/divinylbenzene (PDMS/DVB), are available, but some shortcomings, e.g., fragile needle, deciduous coating, short column lifetime, and insufficient thermal or solvent stability, limit their practical use [8,9,10]. To overcome these, many studies have focused in recent years on metals, such as gold, silver, platinum, titanium, copper, and stainless steel, as substrates for SPME fibers [11]. In addition, a growing trend in developing SPME coating deals with obtaining substrates with high corrosion resistance and high stability as well as high chemical activity and simple surface modification [12,13] (Table 1).

## 3. Preparation Methods for the Analysis of SVOCs or Thermally Stable Compounds

Common SVOCs or thermally stable compounds mostly include pesticides, ERs, carcinogens, such as mycotoxin, process contaminants, among others, which can lead to cancer or impair neurodevelopment in humans. In order to achieve a practical and reliable method, several preparation methods have been developed. Extraction by a solvent is the classic sample preparation technique, which includes liquid–liquid extraction (LLE), soxhlet extraction, solid–liquid extraction (SLE), microwave-assisted extraction (MAE), ultrasonic extraction (USE), accelerated solvent extraction (ASE), and supercritical fluid extraction (SFE) [14,15,16,17,18,19,20,21,22]. Improved extraction methods, such as pressurized liquid extraction (PLE), can increase the diffusion rate and solubility of interferences into the matrix [23,24]. Increasing common clean-up automation and improving instrument design has created a surge in the use of solid-phase extraction (SPE) in a variety of applications has been observed [25,26]. Based on SPE, novel miniaturized SPE methodologies, such as micro-solid phase extraction (MSPE), dispersive-MSPE (DMSPE), matrix solid-phase dispersion extraction (MSPD), and stir bar sorptive extraction (SBSE), have the advantage of requiring low amount of analytes, sorbent, and organic solvents [27,28,29]. Based on miniaturized sorbent-based extraction techniques, several methods have been developed for the analysis of contaminants in real food samples. The development of novel materials, e.g., magnetic molecularly imprinted polymers and other magnetic nanometer materials, with high selectivity to analytes that can at the same time eliminate the interference of the matrix and increase sensitivity and accuracy of the method, is still a promising research field [30,31].

As a general trend, ideal sample preparation techniques should be clean, selective, time-saving, cheap, simple, and environmentally friendly [32]. Compared to MS technology, MS/MS has been shown to accurately detect contaminants in multi-residue analysis and has become an analytical reality for food samples. Thus, in this section, we focused on multi-residue analysis suitable for GC–MS/MS developed in recent years and its matrix effect.

### 3.1. SPE

SPE is mainly based on solid-phase materials acting as sorbents of analytes which are further released under specific conditions. SPE employs a low consumption of organic solvent compared to conventional extraction techniques. However, the steps in the SPE procedure include activation of the SPE column, sample elution, and elution evaporation steps, which implicates a laborious procedure. Moreover, preventing high back pressure is difficult due to the tight packing of the SPE filler. Thus, SPE is combined with other extraction or clean-up procedures, such as LLE and ASE, to obtain more accurate results [33,34,35,36,37,38,39,40].

Based on SPE, the addition of a magnetic adsorbent to the sample with further dispersion with the aid of a vortex, shaker, or sonicator, upon which an external magnetic field is then applied to facilitate efficient retrieval of the magnetic adsorbent particles. Covalent organic frameworks, metal–organic frameworks (MOFs), and molecularly imprinted nanoparticles with uniform morphology offer superior selectivity, large adsorption capacity, and fast binding kinetics that can be used for selective recognition of analytes as well as enrichment and determination of many organic contaminants or pesticide residues [41,42]. Magnetic SPE coupled with GC–MS/MS enables group-selective extractions and detection with enhanced hydrophilicity, dispersibility, adsorptivity, and selectivity, which results in high recovery, precision, and sensitivity of analysis of food samples, being thus a promising alternative for reliable, efficient analysis.

### 3.2. MSPD

As a further development of the SPE method, MSPD is regarded as a promising technique that has been gaining extensive recognition due to the ability to reduce waste of samples and organic solvent. However, the biggest disadvantage of conventional MSPD dispersants (silica gel, C8, C18, etc.) is the lack of selectivity, which may cause interference from non-target substances with similar structures. MOFs, multi-walled carbon nanotubes (MWCNTs), and other newly developed nanomaterials have a high specific surface area and good chemical, mechanical, and thermostability properties and can serve as an adsorbent for enrichment and removal of organic contaminants. These nanomaterials can also be used in MSPD extraction for the pre-treatment of food samples, which have been shown to have short adsorption time, excellent selective recognition, simple operation, low cost, fast extraction efficiency, and low solvent consumption [43].

### 3.3. QuEChERS

The dispersive solid-phase extraction (d-SPE) technique employs acetonitrile extraction partitioning and requires few steps, which reduces the time required to complete the extraction and clean-up procedures [44,45,46,47]. The typical QuEChERS methodology was introduced as a green, user-friendly, and cheap approach to meet the challenge of analyzing trace residue of various organic compounds in foods of plant and animal origin, which are complex matrices. In recent years, the QuEChERS methodology has become increasingly popular as a method to determine contaminants in all kinds of food matrices [48,49,50,51,52,53,54,55,56,57,58,59,60,61,62,63,64,65,66,67,68,69,70,71,72,73,74,75,76,77,78,79,80,81,82,83]. The QuEChERS multi-method is predominantly suitable for the analysis of polar analytes. Target analytes for QuEChERS also include multi-class contaminants, such as pesticide residues, N-nitrosamines, veterinary drug residues, prohibited flavor compounds.

Certain substrates present in foods, such as tea, honeybees, meat, or leek, introduce a heavy matrix interference, which could cause poor peak shape or suppress ionization of analytes or low content target analytes. In this context, further dilution of extracts or the use of a clean-up process, such as USE, multiplug filtration clean-up (m-PFC), robotic clean-up, deep-frozen, SPE methods, are recommended to effectively reduce matrix interference [84,85,86]. The m-PFC method provides a more practical and effective way to perform the clean-up process than the conventional d-SPE method, offering a compromise between the clean-up performance of SPE and the convenience of d-SPE [44]. Since the QuEChERS method is mainly based on the penetration of water and acetonitrile into the sample tissue (at room temperature), if samples have poor water solubility, e.g., beeswax, analysis by the common QuEChERS method might not be efficient [18].

New materials as adsorbents are constantly being developed, among which can be cited tributylamine-functionalized graphene oxide (tri-BuA-rGO), graphitized MWCNTs, and ZrOx cluster [87]. These materials can be used as sorbents in sample preparation since they offer relatively high sensitivity for the analysis of pesticides [67]. Thus, the QuEChERS method is currently a widely used sample preparation method that can be coupled with GC–MS/MS detection and that allows simultaneous determination of a large number of contaminants in a sample.

### 3.4. SBSE

SBSE is a solid-phase extraction technique first described in 1999 [88]. In the past 15–20 years, miniaturized and solventless sample preparation techniques based on sorptive extraction methods, especially SBSE, have gained increasing popularity as simple and environmentally friendly alternatives to the aforementioned conventional methods. A glass-encased magnetic stir bar coated with a polymer, typically PDMS, is employed in this extraction method. In these techniques, extraction and concentration are performed in one step, while the use of a polar PDMS sorbent (which preferentially extracts non-polar compounds) does not require a clean-up process [89]. With SBSE, the sample preparation procedure and the number of solvents used are lower compared with multiple-step extraction procedures that are typically employed for the analysis of persistent organic pollutants (POPs) in solid samples. SBSE coupled with GC–MS has been used in the extraction and analysis of mainly hydrophobic organic compounds in aqueous samples.

### 3.5. Single-Drop Microextraction (SDME)

SDME is an analytical technique that uses only a small amount of water-immiscible solvent for concentrating analytes in aqueous samples. Its advantages include simplicity, low cost, the potential for automation, and the high yield of analytes from the sample matrix. Complex matrices, such as tea, require a step of extraction preparation. Extraction is limited by the partition coefficient of the analytes between the PDMS coating and the sample matrix, as well as by the phase ratio between the PDMS coating and the sample volume [90].

### 3.6. SPME Arrow

The SPME Arrow is a new SPME-based device that combines large sorption phase volumes used in SBSE and the main advantages of the conventional SPME method. The SPME Arrow device consists of a steel rod coated with a sorbent material protected by an outer tube which, together with the arrow-shaped tip, forms the needle. Classical SPME coatings are commercially available for the SPME Arrow. After analytes extraction, the stir bar is removed from the sample matrix and is placed in the thermal desorption unit that extracts analytes from the stir bar. Subsequently, the cooled injection system cryofocuses the desorbed analytes and injects them into the GC–MS/MS system for separation and detection. The SPME Arrow enhances sensitivity since it employs sorption phases that are larger and is hence a more robust technique. Since SPME has emerged only recently, few studies have demonstrated the suitability of this technique in the analysis of different kinds of compounds and samples, which include the analysis of organic compounds and PAHs in water and biogenic VOCs in atmospheric air [91,92].

### 3.7. Other Methods

Directly suspended droplet microextraction was developed to condense contaminants from foods through d-SPE prior to analysis by GC−MS/MS. The extractant is intentionally dispersed into the sample solution in the form of globules through high-speed agitation. This procedure increases the contact area between the binary phases and shortens equilibrium time [90]. The principle is similar to that of the dispersive liquid–liquid microextraction (DLLME) procedure; samples are dissolved into a dispersive solvent (e.g., acetonitrile) and then dissolved in water after being centrifuged and filtered. Lastly, chloroform is used to extract analytes in the DLLME method [93,94] (Table 2).

## 4. Separate, Clean-up, and Derivation Steps

Separate, clean-up, and derivation steps are usually used in further sample purification prior to food contaminants detection.

### 4.1. Gel Permeation Chromatography (GPC)

The GPC clean-up system can be used for the detection of contaminants accumulated in fat or liquid phases of foods. After coextraction of the target analyte and sample fat/liquid, the latter is removed in GPC by the solvent. The GPC clean-up system has been applied in foods of animal origin for further purification and detection of PAHs, chlorinated PAHs, polybrominated diphenyl ethers (PBDEs), and hexabromocyclododecanes (HBCDs) [95,96,97]. GPC has also been used in the combined detection of BaP, benz[a]anthracene (BaA), benzo[b]fluoranthene (BbF), and chrysene (Chr) in grains [98]. The amount of extracted fat depends on the properties of the target analyte, which thus influences the choice of the solvent.

### 4.2. Freezing-Lipid Method

Fat from foods of animal origin usually affects the results of food contaminants detection. Therefore, to separate lipids efficiently, the extract is often deep-frozen, during which a fairly significant amount of non-polar substances are lost [22]. A novel material, namely bond elute enhanced matrix removal (EMR), has a stronger selective adsorption affinity for lipids and has recently been used in the analysis of pesticides in kale, pork, salmon, chicken eggs, and avocado, also used to detect antibacterial drugs in cream disinfection products, specifically adsorbing C5 and long-chain hydrocarbons from lipids [99,100,101].

### 4.3. Derivatization

For single residue or selective residue detection, a derivatization is a useful tool for chromatography analysis which increases extraction rate while reducing detection limit. Especially for analytes with low volatility and high polarity, such as perfluoroalkyl carboxylic acids (PFCAs), PAEs, and 4-methylimidazole, the use of direct GC–MS/MS with selective ion monitoring (SIM) mode without derivatization of samples might not prove a sensitive method for adequate quantification [102].

## 5. Matrix Effect

In the multi-residue analysis of agricultural products, the matrix-induced chromatographic response enhancement, also termed matrix effect (ME), is a major issue that reduces the accuracy and precision of analytical results in food contaminants detection [103]. In general, the term matrix indicates miscellaneous substances that are extracted from samples, and the ME is defined as the direct or indirect alteration or interference in response due to the presence of unintended analytes (for analysis) or other interfering substances in the sample [104]. ME is determined by comparing the slope obtained for the standard calibration curve of the procedure and that of the solvent standard calibration curve, according to the following equation [105,106]:

(1) $$\mathrm{Matrix}\;\mathrm{Effect}\%=(\frac{\mathrm{Slope}\;\mathrm{of}\;\mathrm{calibration}\;\mathrm{curve}\;\mathrm{in}\;\mathrm{matrix}}{\mathrm{Slope}\;\mathrm{of}\;\mathrm{calibration}\;\mathrm{curve}\;\mathrm{in}\;\mathrm{solvent}}-1)\times 100\%$$

Several methods have been proposed to compensate for or overcome ME for various types of matrices, such as matrix-matched calibration, extensive clean-up, dilution, analyte protectant, standard addition method, and stable isotope-labeled pesticides standard mixtures. Dilution is a simple and effective method for reducing ME, but it requires highly sensitive analytical equipment to detect the maximum residue limit of pesticides in foods. Matrix-matched calibration, one of the most popular methods for the multi-residue analysis of agricultural products, is based on the theoretical concept that the ratio of the analytical response to the concentration of a target pesticide must match if the nature of the matrix of both sample and standard calibration solutions is identical. Moreover, internal isotope standards are very expensive and, usually, not available for all substances. Hence, matrix-matched calibration is likely the best strategy to overcome ME and generate highly accurate results [38].

Moreover, a matrix-like effect caused by the presence of pesticides in standard calibration solutions might result in unexpected response alterations and quantification errors, especially when using matrix-free, solvent standard solutions. Many studies suggest that non-polar compounds that have relatively low molecular weights are not susceptible to the matrix-like effect. Small molecules have a shorter residence time in the inlet liner (particularly glass wool), which thereby decreases the probability of ME. Both polarity and stability of a compound are important factors that influence the degree of response alteration caused by the matrix-like effect. It has been shown that highly polar compounds, such as organophosphates, have the potential for high adsorption interaction with active sites, and a fraction of these decompose in the GC system and are susceptible to response alteration induced by the food matrix and analyte protectants. Overcoming and/or compensating for matrix-like effects by improving the GC hardware or using certain food matrices and surrogate compounds (e.g., isotope-labeled pesticide standards) might be important topics for future research [107].

## 6. Emerging Risks

Emerging contaminants may represent a threat to human health and the environment in the future. Such contaminants, such as POPs, BFRs, PFOS, ERs, and other contaminants of unknown biological activity, may have a wide range of physicochemical properties. For instance, POPs are highly stable organic chemicals that resist photolytic, biological, and chemical degradation. POPs might persist in the environment, bioaccumulate through the food chain, and may adversely impact human health and the environment. Among POPs, PAHs, organochlorine pesticides (OCPs), PFCAs, and other compounds in dietary supplement samples can be included [47]. GC–MS/MS has been adopted to accurately quantify these emerging contaminants in order to overcome the low concentration and the complexity of the matrix, which limit the GC–MS/MS analysis [108].

### 6.1. BFRs

BFRs are a group of synthetic chemicals that comprise more than 75 compounds and that have been commercially available since 2003. PBDEs were among the first group of chemicals used worldwide to prevent flammability and have been used as flame retardant additives in a wide array of products, such as plastic materials, fabrics, and furniture. PBDEs comprise over 209 possible congeners produced in three major technical mixtures characterized by different degrees of bromination (penta-BDE, octa-BDE, and deca-BDE). As a consequence, PBDEs can be abundantly found in the environment, wildlife, and human tissue. Novel brominated compounds have been increasingly developed as an alternative to legacy BFRs. OH-PBDEs have oestrogenic, anti-prostagenic, antiandrogenic, and anti-glucocortogenic activity. PBDE congeners have been detected in foods consumed by the general population. Importantly, both PBDEs and OH-PBDEs can alter calcium homeostasis and disrupt intracellular communication [109,110,111]. QuEChERS is a suitable preparation method for PBDEs detection; however, when using other extraction methods, lipids and proteins need to be removed from the sample by using KOH solution/n-hexane partition or GPC process. Lipids and pigments that remain in the extract can be removed using the Florisil cartridge [23,112].

### 6.2. PFOS

PFCAs are a class of PFOS in which hydrogen atoms on the carbon skeleton are completely replaced by fluorine atoms and linked to the carboxylic acid functional groups. The unique physical and chemical characteristics imparted by the fluorinated region of the molecule include water and oil repellency, heat resistance, and surfactant properties, making these compounds suitable for a wide range of industrial and commercial applications. The hardly-degradable characteristic and wide distribution of PFCAs contribute to the persistence of these compounds in the environment and to their bioaccumulation [101]. QuEChERS is the most recommended extraction and separation method for detecting PFCAs [113].

### 6.3. ERs

Many chemical pollutants in the food chain can be considered ERs, which may include certain POPs and their metabolites, pesticides, PAEs, and hormones [114]. PAEs are widely used as plasticizers in various plastic products and packaging and are considered to produce developmental toxicity due to their potential to interfere with hormone homeostasis. Among PAEs, bisphenol A (BPA, 2,2-bis(4-hydroxyphenyl)propane) is used worldwide. QuEChERS, SPE, coupled with solvent extraction methods, are commonly used for PAEs and BPA extraction. Due to the relatively non-volatile and polar nature of PAEs and BPA, derivatization of samples for GC–MS/MS analysis is usually required [115].

PCBs are a group of ubiquitous and organic pollutants with chlorine atoms present in different frequencies and positions on two coupled biphenyl rings. PCBs have been produced for many years and are widely employed in the industry as heat exchange fluids in electric transformers and capacitors, as well as additives in pesticides, paints, sealants, and plastics. Conventional techniques, such as d-SPE or QuEChERS, represent a good choice for the analysis of PCBs in terms of selectivity, accuracy, and precision [116,117].

### 6.4. Mycotoxins

Mycotoxins are secondary toxic metabolites produced by various fungal species (mainly *Aspergillus*, *Penicillium,* and *Fusarium*) and represent an important group of contaminants, particularly in food. Mycotoxins are generally stable, resistant to various processing methods employed in the food industry and could even be found in thermally-processed foods. Several extraction procedures have been reported, such as LLE, SPE, and QuEChERS. However, the QuEChERS method has several advantages over other methods since it is a rapid protocol that requires only small amounts of organic solvents and adequately yields recoveries for all compounds [34,118].

### 6.5. Process Contaminants

Process contaminants are chemicals that are generated when food constituents undergo chemical changes during processing. Prime contaminants, such as acrylamide, PAHs, or furan and furan derivatives, are well-studied. The processes that often introduce PAHs into foods are smoking and grilling [119]. Unrefined plant oils obtained from oilseeds, such as soybeans, rapeseeds, olive seeds, and sunflower seeds, are known to contain high levels of polyaromatic hydrocarbons. PAHs can migrate through the food chain due to their high lipophilicity and accumulate in specific tissues. A new class of PAHs derivatives, known as chlorinated PAHs (Cl-PAHs), have been attracting increasing interest. Emerging Cl-PAHs are also ubiquitous and hazardous pollutants akin to PAHs and other halogenated aromatic compounds, such as polychlorinated dibenzo-p-dioxins, dibenzofurans, and PCBs; the QuEChERS method has been used for sample preparation [120].

Fatty acid esters of monochloropropanediol (MCPDEs) and glycidol (GEs) are emerging process contaminants that are often found in oil-containing products. An SPE clean-up process was used to remove partial glycerides to a certain extent [121]. Ice bath-assisted sodium hydroxide purification can be used for sample extraction for the analysis of ethyl carbamate (EC) and N-nitrosoamines (NAs), which are toxic contaminants found in fermented alcoholic beverages [122].

### 6.6. Contaminants with Unknown Biological Activity

9,10-anthraquinone (AQ) is a new contaminant of unknown sources occurring in tea globally [123]. Moreover, AQ is ubiquitous and currently used as a raw chemical in the paper, pulp, and dye industries. AQ may contribute to the carcinogenic potential of foods. A low level of AQ found in tea plants may be one of the sources of AQ contamination in tea (Table 3).

## 7. Conclusions

This review presents a discussion of current sample preparation methods for the analysis of food contaminants and residues by GC–MS/MS. The main criteria to consider on preparation methods for VOCs analysis, HS extraction, and SPME methods are sensitivity and easiness of quantitation of target analytes. Moreover, GC–MS/MS is gradually being preferred to GC–MS for its higher sensitivity and better separation rather than preparation selectivity. Thus, for the analysis of more SVOCs or thermally stable compounds, it might be advisable to develop methods for the analysis of multiple contaminants of different classes considering a single sample preparation technique and preferably one chromatographic run. Multi-residue preparation methods, such as QuEChERs and adsorption extraction coupled with SBSE and SDME, are the most important technologies currently available, enabling the extraction of as many targets as possible simultaneously. Due to the complexity of certain food matrices, the use of separation, clean-up, and derivatization processes might be useful to increase the extraction rate and reduce the detection limit. In addition, we also proposed guidelines for determining whether ME might occur and interfere with the results; matrix-matched calibration is one of the most popular methods for the multi-residue analysis of agricultural products. Moreover, a discussion of emerging contaminants that may threaten human health and the environment in the future is provided. QuEChERs might be employed to detect these emerging contaminants. In conclusion, a comprehensive strategy is required for sample preparation for the analysis of food contaminants and residues by GC–MS/MS in order to effectively achieve food safety.

## Figures and Tables

**Table 1:** Application of preparation methods for volatile organic compounds (VOCs) analysis in food by GC–MS/MS during the past three years.

Food Groups	Food Matrices	Analyte	Preparation Method		Limit of Detection	Limit of Quantitation	Recoveries	RSD	Ref.
Animal origin food	Beer	Acetaldehyde, acrolein, ethyl carbamate, formaldehyde	Headspace (HS)-Solid-phase microextraction (SPME)		0.03−0.5 μg/L	1.0−2.5 μg/L	90−105%	0.9−12.0%	[8]
	Fish products	6 polycyclic aromatic hydrocarbons (PAHs)	Dynamic HS (DHS) extraction		–	0.01–0.60 ng/g/dw	13−62%	–	[4]
	Grilled meat samples	16 PAHs	SPME		0.02−1.66 ng/L	0.07–5.52 ng/L	85.1–102.8%	2.6–8.5% (intra-day), 4.5–9.4% (inter-day)	[9]
	Aquatic products	Polychlorinated biphenyls (PCBs)	SPME		0.07–0.35 ng/L	−	87.1–99.7%	3.8–9.7%	[10]
	Fish samples	Synthetic musk fragrances	SPME Arrow		0.5–2.5 ng/g	2.5–5 ng/g	−	<23%	[12]
	Seafood species	Benzothiazoles	Subcritical water extraction	SPME	1 and 10 ng/g (dw) for hake, 0.5 and 10 ng/g (dw) for salmon	5–50 ng/g (dw)	2–20%	<21%	[7]
Vegetables	Tomatoes, cucumbers and lettuce	11 Phthalate esters	SPME		0.001–0.430 μg/L	–	>95.2%	<10.8%	[2]
Beverages	Tea	128 Pesticide multi-residue	HS-SPME		–	1–5 μg/kg	70–120%	<20%	[3]
	Liquor, beer, wine, vinegar, tincture	Parabens, phenolic antioxidants, sulfonamide plasticizer, and flame retardant	SPME		0.005–0.2 μg/L	0.01–0.5 μg/L	98–109%	0.8–5.4%	[13]

**Table 2:** Application of preparation methods for semi-volatile organic compounds (SVOCs) analysis in food by GC–MS/MS during the last three years.

Food groups	Food Matrices	Analyte	Preparation Method		Limit of Detection	Limit of Quantitation	Recoveries	RSD	Ref.
			Extraction	Clean-up					
Animal origin food	Catfish	219 Pesticides and metabolites (178 pesticides and 41 environmental contaminants)	QuEChERs	SPE	<50 ng/g for 90% analytes	1–20 ng/g	70−120% for 80% analytes	<20% for 80% analytes	[33]
	Fish, shrimp and shellfish	Organochlorine pesticides (OCPs) and polychlorinated biphenyls (PCBs)	Matrix solid-phase dispersion (MSPD)		0.011–0.046 ng/g	0.037–0.153 ng/g	70–120%	<20%	[28]
	Bivalve shellfish samples	Amide/Dinitroaniline/Substituted Urea Herbicides	QuEChERS		–	0.3–8.88 μg/kg	81–109%	<8%	[51]
	Egg (egg white, egg yolk, and whole egg)	Dinitolmide residue and its metabolite	ASE		0.8–2.8 μg/kg	3.0–10.0 μg/kg	>80%	2.96–5.21% (intra-day) 3.94–6.34% (inter-day)	[20]
	Chicken eggs	80 Pesticides	Acetonitrile with 5% formic acid	Bond elute enhanced matrix removal-lipid	0.02−9.725 µg/kg	0.066–30.261 µg/kg	65.3–124%	4.3−24%	[32]
	Poultry egg (whole egg, albumen and yolk)	Spectinomycin and lincomycin	ASE	SPE	2.3−4.3 μg/kg	6.0−9.5 μg/kg	80.0−95.7%	1.0−3.4%	[34]
	Meat (chicken, pork, and beef) and fish (catfish and salmon)	Organophosphate esters (OPEs)	QuEChERS	Automated robotic clean-up	–	0.5−1 ng/g	70−120%	≤20%	[86]
	Chicken tissues	Dinitolmide and its metabolite	ASE	SPE	0.8–2.5 μg/kg	2.7–8.0 μg/kg	81.96–94.31%	1.72–5.37%	[35]
	Meats and poultry	200 pesticides and 65 environmental contaminants	QuEChERS	SPE	–	<5 ng/g (for 90% analytes)	70–120%	≤20%	[36]
	Raw propolis	14 Lipophilic pesticides	n-hexane	SPE	–	0.002–0.020 μg/g	61.0–106.8%	≤ 16.9%	[37]
	10 Beeswax samples	160 Pesticides	Acetonitrile–ethyl acetate (1:3, *v*/*v*)		–	<20 μg/kg	80−110% for most analytes	<8% for most analytes	[18]
	Honeybees	Pesticide residues	USE	QuEChERS	–	5 μg/kg	70–120%	<20%	[84]
	122 Honey samples	53 Pesticide residues	QuEChERS			0.001–0.01 mg/kg	70.0–120.0%	≤20%	[52]
	Organic honeys	POPs, pesticides and antibiotic residues	QuEChERS		–	7.15–9.80 ng/g	82–120%	<20%	[53]
	Bovine milk	78 Drugs and 238 pesticides	QuEChERS		−	0.1–10 ng/g	70–120%	<20%	[54]
	Hen eggs	60 Pesticides	QuEChERS		0.001–0.004 mg/kg	<10 µg/kg for 83% analytes	70–120%	<20%	[50]
	Porcine meat	39 Pesticide residues	QuEChERS	Rapid multiplug filtration clean-up (m–PFC)	–	0.01 mg/kg except pyrimethanil	74–118% except pyrimethanil	1−16%	[85]
Grain, Vegetables and fruits	207 Vegetable samples	10 New–generation pesticides	MAE		1.4–3.6 ng/g	–	80–111%	<11%	[21]
	249 Grain, beans, fruit and vegetables samples	365 Pesticide residues	acetonitrile	SPE	0.0001–0.0414 mg/kg	0.0002–0.1367 mg/kg	70–120% for 95% analytes	<20%	[39]
	Mangoes	113 Pesticides	QuEChERS		<4 μg/kg	<10.0 μg/kg	70–120%	<20%	[55]
	Pigeonpea grains	79 Pesticides	QuEChERS		0.53–3.97 µg/kg	1.60–10.05 µg/kg	70–120%	<15%	[48]
	Apples; mangos; strawberries; cucumbers and tomatoes	41 Triazines and pyrethroids residues	QuEChERS		0.03 − 10.22 μg/kg	–	–	–	[56]
	Fruits (apple and grapes) and vegetables (apple, grapes, cauli–flower, cabbage, peas, potato)	Cypermethrin, chlorpyrifos, methyl parathion, ethion, captan, malathion, and triazophos	QuEChERS		0.0011–0.012 μg/kg	0.0012–0.035 mg/kg	94–99%	3.3–8.1%	[57]
	69 Fruits and vegetables samples	203 Pesticides	QuEChERS		–	2 μg/kg	70–120% in tomato, apple, and orange for 97% compounds. low recoveries for orange	<20% except for biphenyl, butylate, chlozolinate, and pyrifenox; <20% (inter-day) for 97% analytes	[58]
	Chinese vegetables and fruits	Pyrethroid pesticides	QuEChERS		0.3–4.9 μg/kg	~10 μg/kg	78.8–118.6%	<14.8%	[59]
	Tomato	9 Dinitroaniline herbicides	Vortex-assisted dispersive liquid–liquid microextraction (VA-DLLME)		0.3–3.3 µg/L	2–10 µg/kg	64.1–87.9%	≤15.1% (inter-day)	[90]
	Tomatoes	20 Pesticides	QuEChERS		2.6–31.3 μg/kg	6.9–93.8 μg/kg	72.5–119.7%	1.17–14.62%	[60]
	Garlic, onion, and sugar beet	Total ethofumesate residues	SPE		0.0005 mg/kg	0.01 mg/kg	94−113%	1.8−5.7%	[61]
	Vegetables	14 Pyrethroids	QuEChERS		–	2–10 μg/kg in tea, 2 μg/kg In tomato, pear, and zucchini	–	–	[62]
	16 Common bean samples	142 Pesticide residues	QuEChERs		–	20–100 µg/kg	70−120% for 61.4% analytes	<20% for 61.4% analytes	[63]
	211 Vegetable samples	12 Pesticide residues	QuEChERS		0.0005–0.0023 mg/kg	0.0009–0.0047 mg/kg	74–120% at 0.01 mg/kg, 75–123% mg/kg	2–9% at 0.01 mg/kg, 0.5–16 mg/kg	[64]
	Greenhouse strawberries	16 Pesticide residues	QuEChERS		0.1–0.8 µg/kg	0.3–2.8 µg/kg	80.7–117.2 µg/kg	0.6–14.6%	[65]
	Dried fruits	38 Multi-class pesticides	QuEChERS		–	0.02–5 µg/kg	70–120%	<20% for 92% samples	[66]
Beverages	Tea	Pyrethroid insecticides	magnetic SPE		0.0065–0.1017 µg/L	–	–	<9.7% (intra-day), <11.95% (inter-day)	[31]
	Chinese liquor and liquor–making raw materials (sorghum and rice hull)	124 Pesticide residues	d–SPE		0.00003–0.015 mg/kg	0.0001–0.05 mg/kg	71–121%	<16.8% except cyprodinil, di-flufenican and prothioconazole	[44]
	Tea	131 Pesticides	d-SPE		0.5–5.0 µg/kg	1.5–16.7 µg/kg	78.2–113.9%	<15.8%	[45]
	Tea	11 Pesticides	d-SPE		0.10–2.10 µg/kg	0.29–6.20 µg/kg	73.4–106.4%	1.9–6.6% (within-run precision), 12.1% (between-run precision)	[46]
	38 Tea samples	45 Pesticide residues	d-SPE	Speed-regulated directly suspended droplet microextraction (SR-DSDME)	–	0.1−47 µg/kg	70–120%	<20%	[90]
	Green tea	203 Pesticide residues	QuEChERS	SPE	0.33–16.67 μg/kg	1–50 μg/kg	70−120% for most analytes	<20% for most analytes	[38]
Oil	Soybean	203 Pesticides	SPE		–	<0.01 mg/kg	70–120%	<20%	[25]
	Edible oils	Organophosphorus pesticide residues (OPPs)	QuEChERS		0.16−1.56 ng/g	0.61−5.00 ng/g	81.1−113.5%	<8.2 (intra–day) <13.9% (inter–day)	[67]
	Edible oils	Pesticides	LLE	Enhanced matrix removal (EMR)-lipid cartridge	–	1 ng/g	70–120%	<20%	[22]
Sugar	Sugarcane	Fipronil and its metabolites	QuEChERS		0.0015–0.002 µg/g	0.005 µg/g	80.7–98.5%	1.80–12.81% (intra–day), 1.2–16.5% (inter–day)	[68]
Medicinal plants	*Lycium barbarum* (goji)	6 Active ingredients of pyrethrins	SPE		0.24–2.1 µg/kg	0.8–7 µg/kg	88.3–111.5%	0.4–8.3%	[26]
	Panax notoginseng (Burk) F.H.Chen root	Pesticide residues	QuEChERS		0.0015 mg/kg	0.005 mg/kg	94–125% for quintozene, 84–119% for hexachlorobenzene (HCB)	6.2–16.1%	[69]
	Chenpi	133 Pesticide residues	QuEChERS		–	0.005–0.01 mg/kg	70–112.2%	0.2–14.4%	[70]
	*Notoginseng Radix et Rhizome*	116 Pesticide residues	QuEChERS		–	0.01–0.05 mg/kg	64.3–119.4%	<18.3%	[71]
	Dried Herbs	235 Pesticides	QuEChERs		0.0003–0.0007 mg/kg	0.001–0.002 mg/kg	70–120%	<20%	[72]
	Herbal species–ready application	201 Pesticides	QuEChERS		–	≤10 ng/mL	70.0–120.0%	≤20%	[73]
	Cardamom	243 Pesticide residues	QuEChERS		–	10 mg/kg	70.0–120%	<20%	[74]
Condiment	Capsicum annum	Chlorantraniliprole	QuEChERS		0.005 mg/kg	0.01 mg/kg	85–91%	<2% (intra-day and inter-day)	[76]
	Pepper, chili peppers and its sauce product	47 Pesticide residues	QuEChERS		–	0.01 mg/kg	70–120% (except for pyrimethanil)	<17%	[77]
Edible fungi	Edible mushrooms	10 Pyrethroid insecticides	QuEChERs		0.015−1.67 μg/kg	0.051−5.57 μg/kg	72.8–103.6%	<13%	[78]
Condiment and medicinal plants	Spices and herbs	140 Organic contamination	QuEChERS		0.04–5.20 ng/g	0.08–17.19 ng/g	80–137%	<20%	[79]
Muti-matrices	Tea and herbal infusion	300 Pesticides	QuEChERS		0.018–40 µg/kg	0.06–135 µg/kg	70–120%	<20%	[80]
	Dried herbs and dried fruit	236 Pesticides	QuEChERS		0.001 mg/kg	0.005 mg/kg	62–125%	1–19%	[81]
	Beverages, ‘pesto’ sauces, meat preparation	Methyleugenol	QuEChERS		0.4 μg/kg	1 μg/kg	94.29–100.27%	<9%	[82]
	Beef jerky, cod liver oil, candy	8 Prohibited flavor compounds	QuEChERS		0.005−0.2 μg/kg	0.03−0.8 μg/kg	80.2–110.6% (beef jerky), 82.3–94.1% (cod liver oil), 83.6–104.1% (candy)	−	[83]

**Table 3:** Application of preparation methods for emerging risks analysis in food by GC–MS/MS during the past three years.

Food Type	Sample	Analyte	Preparation Method		Limit of Detection	Limit of Quantitation	Recoveries	RSD	Ref.
			Extraction	Clean-up					
Animal origin food	Fish	Persistent organic pollutants (POPs)	Accelerated solvent extraction (ASE)		–	0.01–4.44 ng/g	70–120%	<20%	[19]
	Fish	POPs	Hexane–acetone	Florisil and silica gel	0.001–0.040 ng/g	0.004–0.12 ng/g	60–127%	≤20%	[17]
	Sea fish	Dihydroxylated PBDEs	Pressurized liquid extraction (PLE)	Florisil cartridge	3.98–38.74 pg/g	11.95–116.22 pg/g	19–101% in 10 ng, 28–88% in 20 ng, 42–90% in 40 ng	–	[23]
	77 Smoked meat products	PAHs	Extracted by dichlormethane/hexane	Gel permeation chromatography (GPC)	0.02–0.03 µg/kg	0.06–0.09 µg/kg	97–115%	3–9% (intra-day), 6–9% (inter-day)	[95]
	Fish shellfish and muscle of terrestrial animals	PBDEs and hexabromocyclododecanes (HBCDs)	QuEChERS	GPC	–	10 pg/g, for BDE-206; 100 pg/g for BDE-209	72–97%	9–22%	[96]
	5 Kinds of marine products	9 Pefluoroalkyl carboxylic acids (PCAs)	Alkaline digestion	SPE	0.04–0.10 ng/g	–	54.72−107.29%	1.53−11.89%	[100]
	233 Fish and aquatic invertebrate samples	6 Polychlorinated biphenyls (PCBs)	QuEChERs		3–13 ng/g	9–40 ng/g	75–113%	2–12%	[116]
	Mussels and clams	PCBs, Polybrominated diphenyl ethers (PBDEs), organochlorine pesticides (OCPs), PAHs, and perfluoroalkyl substances (PFASs)	QuEChERS		–	0.5–5 ng/g	70–120%	<20%	[108]
	Marine bivalves	211 Analytes, including pesticides, PCBs, PAHs, PBDEs, and other flame retardants	QuEChERS		–	0.2–10 μg/kg	80–120%	–	[109]
	Shellfish samples	84 PCBs and OCPs	QuEChERS		0.004–2.705 μg/kg	0.01–9.02 μg/kg	70–120%	<10%	[117]
	Pork	PBDEs and PFASs	QuEChERS		5–50 pg/g	15–150 pg/g	80–119%	6–19% (intra-day), 9–20% (inter-day)	[112]
	Plastic packaged baby food samples	Bisphenols (BPs)	Liquid extraction	Dispersive sorbents	0.1–1 ng/g	0.5–4 ng/g	91–110%	<13%	[114]
	Chicken meat and edible offal	8 Trichothecenes	QuEChERS		0.05–0.15 μg/g	0.25–0.75 μg/kg	85.1–108.4%	<8%	[118]
	60 Infant formula and baby foodproducts	Monochloropropanediol (MCPDEs) and glycidol (GEs)	SPE		0.1−0.6 µg/kg	1, 2, and 1.2 μg/kg in baby food; 1.2, 1, and 0.5 μg/kg in infant formula	91−106% for baby food; 94−99% for infant formula	1.2−7.8%	[121]
	Milk and milk powder	Sodium fluoroacetate (1080)	SPE		0.0013–0.0025 µg/kg	0.0042–0.0085 µg/kg	90–105%	<6%	[40]
	Milk	Hexamethylenetetramine (HMT)	Magnetic molecularly imprinted polymers		0.3 μg/kg	1.0 μg/kg	88.7–111.4%	2.6–5.2% (intra-day), 3.6–11.5 (inter-day)	[30]
Grains, vegetables and fruits	Wheat flour samples	Bifenox, dichlobenil and diclofop methyl	MSPE		0.39 ng/g (DCB), 0.24 ng/g (BFO), 0.68 ng/g (DCM)	1.33 ng/g (DCB), 0.76 ng/g (BFO), 2.18 ng/g (DCM)	88.8–96.6%	<3.5%	[27]
	Cereal products	Sum of BaP, benz[a]anthracene (BaA), benzo[b]fluoranthene (BbF), and chrysene (Chr)	Extracted dichlormethane/hexane (1:1, *v*/*v*)	GPC	0.002–0.006 μg/kg	0.07–0.75 μg/kg	92–103%	4–19%	[97]
	12 Commercially available plant extract-based dietary supplement samples	21 POPs	Stir-bar sorptive extraction (SBSE)		–	0.00899−0.0931 ng/g	–	4.48−12.9%	[89]
	Vegetables	17 Emerging contaminants	Ultrasound-assisted matrix solid-phase dispersion (UAE-MSPD)		0.1–0.4 ng/g	0.1–0.8 ng/g	55–138%	≤13% (intra-day), ≤16% (inter-day)	[47]
	Tomatoes	20 Organochlorine pesticides	QuEChERS		0.001–0.1 μg/kg	0.01–0.33 µg/kg	71.2–95.3%	<20%	[113]
	Carrots, turnips and potatoes	Bisphenol A, its chlorinated derivatives and structural analogues	Focused ultrasound solid–liquid extraction	Dispersive solid-phase extraction (d-SPE)	0.02–0.33 ng/g^/^dw	0.05–1 ng/g^/^dw	74–105 %	<12%	[115]
Condiment	Capsicum cultivars	12 Brominated flame retardants (BFRs)	QuEChERS		1.4–9.3 μg/kg	4.6–30.9 µg/kg	66–104%	<20%	[110]
Oils	Edible Oils	PAHs	d-SPE		0.06–0.21 μg/kg	0.19–0.71 μg kg^−1^	98–108%	2–5% (intra-day), 4–6% (inter-day)	[49]
	Edible Oils	PAHs	d-SPE		0.06–0.21 μg/kg	0.19–0.71 μg/kg	98–108%	2–5% (intra-day), 4–6% (inter-day)	[107]
	Fish oils	BFRs and organochloride pollutants	Vortex assisted liquid–liquid microextraction (VALLME) technique		0.2–0.7 ng/g	–	76–90%	<20%	[111]
Beverages	30 Tea samples	38 PCBs	d-SPE		0.1–2.9 μg/kg	2.0–10 μg/kg	73−113%	5−20%	[49]
	54 Beverages	Gamma-hydroxybutyrate (GHB)	Dispersive liquid–liquid microextraction (DLLME)		0.5 ng/mL	–	78.2−84.7%	4.9−5.7%	[93]
	Beverage samples	15 PAEs	SPE		0.005–2.748 µg/L	0.018–9.151 µg/L	79.3–121.8%	<8.8% (intra–day),<9.9% (inter-day)	[15]
	Brazilian Cachaça	93 Pesticides and 6 PAHs	QuEChERS		2.5 µg/L	10.0 µg/L	86.7–118.2%	≤20%	[120]
	Yellow rice wine	Ethyl carbamate (EC) and N-nitrosoamines (NAs)	Ice bath-assisted sodium hydroxide purification		0.1–0.5 μg/kg	0.5–1.5 μg/kg	81.5–121%	2.2–9.4% (intra-day), 1.6–7.9% (inter-day)	[122]
	Tea	9,10-Anthraquinone (AQ)	Solvent extraction		10 μg/kg (tea shoots, tea), 0.4 μg/L (tea brew)	0.01 mg/kg (tea shoots, tea), 0.4 mg/kg (tea brew)	87.0–110.8%	2.3–14.6%	[123]
Medicinal plants	Ginseng	5 Organochlorine pesticide	LLE		–	0.02–0.12 µg/L in liquid samples, 0.001–0.004 μg/kg in solid samples	70.3–85.6% in liquid samples, 83.4–106.9% in solid samples	–	[14]
Muti-matrices	Spices and dried herbs	PAHs	SPE		0.25 μg/kg	0.5 μg/kg	close to 100%	<22%	[119]
	Cow milk, plastic bottled beverage, and edible oil	PAEs	SPE		0.15–1.64 ng/g	–	73.7–98.1%	1.7–10.2%.	[41]
Food Packaging Materials	Food Packaging Materials	20 PAEs	Solvent extraction		1.7–62.5 µg/kg	5.5–208.3 µg/kg	82.1–110.8%	0.3–9.7%.	[42]
